# Alpha-Toxin Contributes to Biofilm Formation among *Staphylococcus* *aureus* Wound Isolates

**DOI:** 10.3390/toxins10040157

**Published:** 2018-04-16

**Authors:** Michele J. Anderson, Emily Schaaf, Laura M. Breshears, Heidi W. Wallis, James R. Johnson, Christine Tkaczyk, Bret R. Sellman, Jisun Sun, Marnie L. Peterson

**Affiliations:** 1 Experimental and Clinical Pharmacology, College of Pharmacy, University of Minnesota, Minneapolis, MN 55455, USA; manderson10@mmm.com (M.J.A.); bresh006@umn.edu (L.M.B.); wang1530@umn.edu (H.W.W.); 2 Heath Care Business Group, 3M, St. Paul, MN 55144, USA; 3 Department of Pediatric Infectious Diseases, Medical School, University of Minnesota, Minneapolis, MN 55455, USA; scha1123@umn.edu; 4 Veterans Affairs Medical Center, Minneapolis, MN 55417, USA; johns007@umn.edu; 5 Department of Medicine, Medical School, University of Minnesota, Minneapolis, MN 55455, USA; 6 Department of Infectious Disease, MedImmune, Gaithersburg, MD 20878, USA; tkaczykc@medimmune.com (C.T.); sellmanb@medimmune.com (B.R.S.); 7 Department of Veterinary Population Medicine, College of Veterinary Medicine, University of Minnesota, St. Paul, MN 55455, USA; jisun.haan@state.mn.us; 8 Infectious Disease Laboratory, Minnesota Department of Health, St. Paul, MN 55164, USA; 9 School of Pharmacy, The University of Wyoming, Laramie, WY 82071, USA

**Keywords:** toxin, bacterial toxin, biofilm, *Staphylococcus aureus*, wound

## Abstract

Biofilms complicate treatment of *Staphylococcus aureus* (SA) wound infections. Previously, we determined alpha-toxin (AT)-promoted SA biofilm formation on mucosal tissue. Therefore, we evaluated SA wound isolates for AT production and biofilm formation on epithelium and assessed the role of AT in biofilm formation. Thirty-eight wound isolates were molecularly typed by pulsed-field gel electrophoresis (PFGE), multilocus sequence typing (ST), and *spa* typing. We measured biofilm formation of these SA isolates in vitro and ex vivo and quantified ex vivo AT production. We also investigated the effect of an anti-AT monoclonal antibody (MEDI4893*) on ex vivo biofilm formation by methicillin-resistant SA (USA 300 LAC) and tested whether purified AT rescued the biofilm defect of *hla* mutant SA strains. The predominant PFGE/ST combinations were USA100/ST5 (50%) and USA300/ST8 (33%) for methicillin-resistant SA (MRSA, *n* = 18), and USA200/ST30 (20%) for methicillin-susceptible SA (MSSA, *n* = 20). Ex vivo AT production correlated significantly with ex vivo SA wound isolate biofilm formation. Anti-alpha-toxin monoclonal antibody (MEDI4893*) prevented ex vivo biofilm formation by MRSA USA300 strain LAC. Wild-type AT rescued the ex vivo biofilm defect of non-AT producing SA strains. These findings provide evidence that AT plays a role in SA biofilm formation on epithelial surfaces and suggest that neutralization of AT may be useful in preventing and treating SA infections.

## 1. Introduction

Biofilms are complex microbial communities embedded in extracellular matrix that are resistant to antimicrobial treatment and host immune responses [1]. Several studies have confirmed the presence of biofilms that contain *Staphylococcus aureus* (SA) in wounds and skin and soft tissue infections (SSTIs) [2,3,4]. SA has been isolated from up to 62% of SSTIs in military and veteran populations [5,6]. While numerous SA virulence factors have been described, the relationships among SA clonal background, virulence factor production, and biofilm formation are currently unknown for SA wound isolates. 

Methicillin-resistant SA (MRSA) USA300 is the predominant pulsed-field gel electrophoresis (PFGE) type of SA associated with SSTIs in the United States [7,8]. The heightened virulence of community-associated MRSA USA300 in experimental models has been associated with the production of alpha-toxin (AT), a 33 kDa pore-forming, cytolytic exotoxin [9]. Alpha-toxin is cytotoxic to diverse host cells, including immune cells, endothelial cells, and epithelial cells [10,11,12,13]. Alpha-toxin is also expressed by most MSSA and MRSA isolates. A study of 994 respiratory SA isolates from 34 countries determined that the AT gene, *hla*, was present and Hla expressed by 99% of MSSA and 83.2% of MRSA isolates regardless of geographic region [14]. 

Previously, we determined that AT contributed to ex vivo mucosal biofilm formation by MSSA USA 200 isolates in a AT dependent manner. Furthermore, AT role in biofilm formation was context-dependent and was necessary for biofilm formation on ex vivo mucosa (a biotic surface) but not in vitro on polystyrene (an abiotic surface) [15]. Similarly, a previous study detected AT in all biofilms formed by SA isolates in a three-dimensional Leiden human epidermal model, but in only, some of the biofilms produced by the same strains on polystyrene [16]. 

This study was undertaken to explore whether AT could be a contributing factor to SA wound-associated biofilms. First, we molecularly characterized a collection of clinical SA wound isolates (20 MSSA and 18 MRSA) obtained from the Minneapolis Veterans Affairs Medical Center (MVAMC) in 2013. We hypothesized that SA wound isolates would form biofilms, particularly in a wound-like environment (an epithelial surface), and that biofilm formation ex vivo depends on the amount of AT produced. We tested these hypotheses by comparing the ability of the SA clinical wound isolates to form a biofilm in vitro on polystyrene versus ex vivo on mucosal explants and by assessing whether AT production correlated with ex vivo biofilm formation. We also determined the ability of MEDI4893*, an anti-AT monoclonal antibody (mAb), to inhibit ex vivo mucosal biofilm formation by a representative USA300 MRSA strain (LAC). Finally, we compared the ability of wild-type (WT) AT and a cell binding (but non-cytolytic) single-amino-acid AT mutant (H35L) to rescue the ex vivo biofilm defect of select wound SA isolates that do not produce AT. Our findings provide evidence that AT plays a role in SA biofilm formation and suggest that neutralization of AT may be useful in preventing and treating SA wound infections.

## 2. Results

### 2.1. Strain Genotyping

Thirty-eight SA wound isolates (18 MRSA and 20 MSSA) from the MVAMC (May through July 2013) were analyzed by PFGE, MLST, and *spa* typing (Table 1). The MRSA isolates represented predominantly PFGE types USA100 (10, 56%) and USA300 (6, 33%), only two were non-typeable by PFGE. The MRSA isolates represented four STs, predominantly ST5 (10, 56%) and ST8 (6, 33%), plus one each of ST231 and ST88. The two most common PFGE type/ST combinations were USA100/ST5 (9, 50%) and USA300/ST8 (6, 33%). Six *spa* types were represented; t002 was most common (8, 44%), followed by t008 (6, 33%). The MSSA isolates were more genetically diverse. They represented six different PFGE types and 10 STs. Eight (40%) were PFGE non-typeable. The most common PFGE type/ST combination was USA200/ST30 (4, 20%). Similarly, MSSA isolates represented 12 different *spa* types, most commonly t012 (4, 20%). 

### 2.2. Ex Vivo AT Production on PVM Explants 

**Figure 1:**
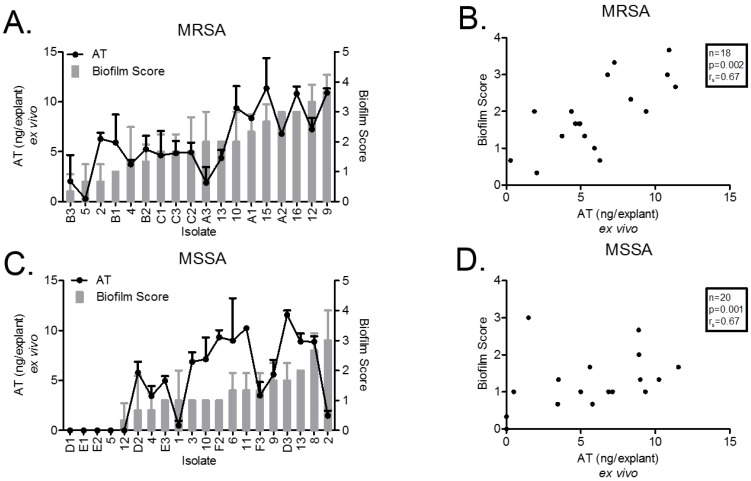
**Ex vivo biofilm formation and alpha-toxin production.** The degree of biofilm formed on porcine vaginal mucosa (PVM) explants was highly variable. (**A**) All methicillin-resistant *S. aureus* (MRSA) isolates formed biofilms; scores ranged from 0.67 to 3.67 (grey bars, right axis). Similar to the MRSA ex vivo biofilms, alpha-toxin (AT) was highly variable, ranging from <1.0 ng to >10 ng per explants (black circles, left axis). (**C**) In contrast, several methicillin-susceptible *S. aureus* (MSSA) isolates did not form biofilms on the PVM; overall, the scores were lower than for MRSA (grey bars, right axis). AT was not detected by ELISA (<60 pg/mL) in MSSA isolates that failed to form biofilm (black circles, left axis). Increased ex vivo alpha-toxin production corresponds with ex vivo biofilm formation (black circles, left axis). (**B**,**D**) Correlation analysis of ex vivo alpha-toxin production vs. ex vivo biofilm formation showed a strong direct correlation for both MRSA (*n* = 18, r_s_ = 0.67, *p* = 0.002) and MSSA (*n* = 20, r_s_ = 0.67, *p* = 0.001).

After a 72-h incubation on PVM explants, AT production by all SA isolates was quantified by sandwich ELISA (Figure 1). All SA isolates reached a similar total bacterial density on the explants at 72-h (~1 × 10^8^ CFU/explant, Figure S1) The MRSA USA300/ST8 isolates produced significantly more AT than MRSA USA100/ST5 isolates (9.35 ± 0.94 ng/explant [*n* = 6], versus 4.52 ± 0.77 ng/explants [*n* = 9], respectively; *p* = 0.002) (Figure 1A). Five MSSA isolates (25%: D1, E1, E2, 5, and 12) did not produce detectable AT, and four of these were ST30 (Figure 1C). ST30 MSSA strains are known to have a high frequency of a nonsense point mutation (CAG [Gln] to TAG [stop]) at codon 113 in the AT-encoding gene *hla*, which attenuates AT production [17,18]. Therefore, we sequenced a region of *hla* (position 1 to 873 bp) that spans codon 113 in all 20 MSSA isolates and identified the mutation in six of the isolates (1, 5, 12, D1, E2, and E3), all of which were ST30 (Table 1).

### 2.3. Ex Vivo Biofilm Production on PVM Explants

**Figure 2:**
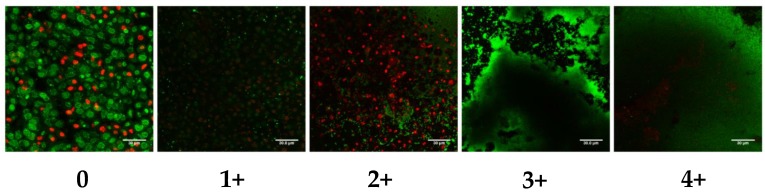
**Scoring system for *S. aureus* biofilm images.** Biofilm formation by *S. aureus* (SA) isolates on biological substrate (porcine vaginal mucosa (PVM) explants) at 72 h was evaluated by LIVE/DEAD^®^ staining (SYTO^®^ 9 [green = live] plus propidium iodide [red = dead]), which does not discriminate eukaryotic from prokaryotic cells, and confocal laser scanning microscopy. Ex vivo biofilms were scored on a 5-point scale. **0:** no biofilm, few if any attached bacteria, live epithelial cells observed throughout image (large, green, punctate staining) with some interspersed dead cells (red punctate staining). **1+**: biofilm covers >0–25% of surface area; live, adherent bacteria observed as small, bright green punctate staining interspersed among live (green) and dead (red) epithelial cells. **2+**: biofilm covers 26–50% of surface area; microcolonies observed as clusters of live bacteria in the lower right quadrant, more mature biofilm observed in the upper right quadrant (continuous green staining), many dead (red) epithelial cells apparent, some of which have sloughed off (black areas). **3+**: biofilm covers 51–75% of surface area; large, cloudlike, live (green) biofilm observed and no epithelium evident. **4+**: biofilm covers 76–100% of surface area; thick, dense biofilm (large proportion of image covered in green, small red punctate staining in the middle to lower left quadrant are dead bacteria). Isolates (*n* = 3) individually scored by two blinded observers. This was done twice for a total of 6 observations per isolate. Scale bar within each image is equal to 30 μm.

We evaluated biofilm formation by SA isolates on a biological substrate (PVM explants) with LIVE/DEAD^®^ staining and CLSM with the scoring system illustrated in Figure 2. In two previous studies, we described the development of this method and determined the kinetics of MSSA and MRSA biofilm formation from adherence at 24 h, microcolony formation at 48 h to mature biofilm at 72 h [15,19]. Therefore, the scoring system was based on the 72 h (mature biofilm time point). 

All 18 (100%) MRSA isolates and 16 (80%) of 20 MSSA isolates produced some biofilm (Figure 1A,C). The remaining 4 MSSA isolates (D1, E1, E2 and 5) that did not form a biofilm also did not produce detectable AT. AT production and biofilm formation were strongly correlated for both MRSA (r_s_ = 0.67, *p* = 0.002) and MSSA (r_s_ = 0.67, *p* = 0.001) isolates (Figure 1B,D). The relationship between biofilm formation and AT production was largely linear except for two isolates. MRSA isolate 2 produced AT (mean 6.28 ng/explant) but minimal biofilm (mean biofilm score of 0.67). MSSA isolate 2 produced a low amount of AT (mean 1.48 ng/explant) but formed a biofilm (mean biofilm score of 3). 

### 2.4. In Vitro Biofilm Production

**Figure 3:**
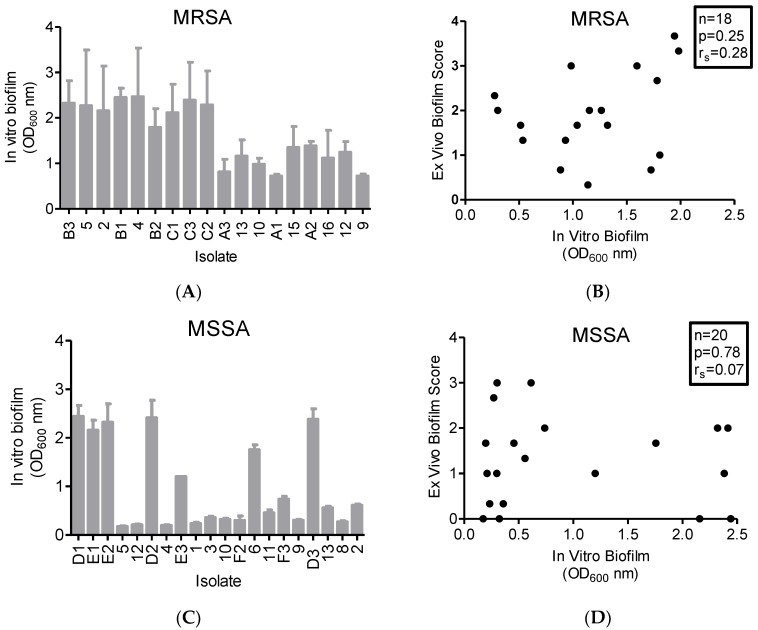
**MRSA and MSSA isolates form biofilm in vitro (*n* = 8 per isolate).** (**A**) OD_600_ of crystal violet-stained methicillin-resistant *S. aureus* (MRSA) *n* = 18, and (**C**) methicillin-susceptible *S. aureus* (MSSA) *n* = 20 biofilms following 24 h incubation (grey bars). (**B**) We performed a correlation analysis with Spearman correction of in vitro vs ex vivo biofilm formation. There was a weak correlation between microtiter biofilm density and degree of porcine vaginal mucosa biofilm for MRSA (*n* = 18, r_s_ = 0.28, *p* = 0.25). (**D**) For MSSA isolates, there was a very weak correlation between the in vitro biofilms and those grown on a biological substrate (*n* = 20, r_s_ = 0.07, *p* = 0.78).

All 18 MRSA isolates were strong or very strong biofilm formers (OD > 0.24) at 24 h in glucose-supplemented broth in polystyrene wells. However, the extent of biofilm production varied markedly, where four isolates (22%) produced biofilms with OD > 0.24 to <1.0, and the remaining 14 isolates (78%) produced biofilms with OD > 1.0 (Figure 3A). In contrast, of the 20 MSSA isolates four (20%) [7,10,20,21] were weak biofilm formers (OD > 0.12 to <0.24, Figure 3C). The remaining 16 MSSA isolates were strong or very strong biofilm formers (OD > 0.24), including seven (35% of 20) that produced very strong biofilms (OD > 1.0). Correlation analysis of in vitro versus ex vivo biofilm production showed a weak correlation for MRSA isolates (r_s_ = 0.28, *p* = 0.25, Figure 3B) and a very weak correlation for MSSA isolates (r_s_ = 0.07, *p* = 0.78, Figure 3D). Notably, MSSA strains that produced no detectable AT and did not form a biofilm on the ex vivo mucosa (strains D1, E1, and E2) were very strong biofilm formers in vitro. 

### 2.5. Neutralization of AT Prevents MRSA Biofilm Formation on Mucosal Tissue

**Figure 4:**
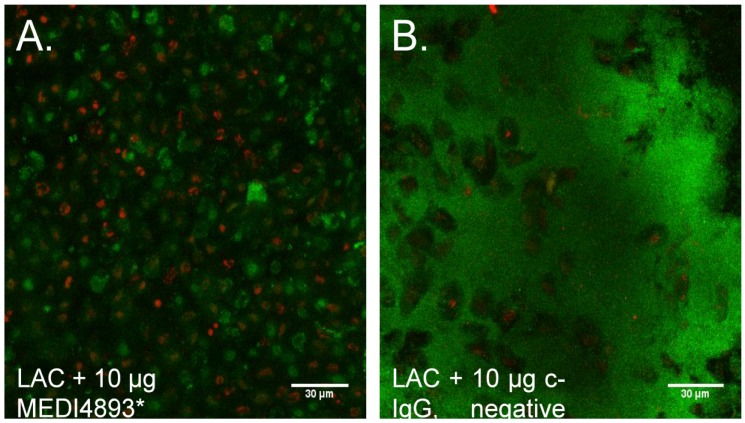
**Biofilm formation is prevented by anti-alpha-toxin monoclonal antibody.** Porcine vaginal mucosa (PVM) explants were pretreated with 10 uL of MEDI4893* (MedImmune, Fredricksburg, MD, USA) or isotype control antibody (c-IgG) for 1 h prior to infection. Three days post-infection, explants were stained and imaged by confocal laser scanning microscopy. (**A**) MEDI4893* completely abrogated MRSA’s ability to form a biofilm on PVM epithelium. (**B**) C-IgG-treated MRSA developed a 4+ biofilm. Scale bar equals 30 μm.

Our findings that SA AT production correlated with ex vivo biofilm formation was supported by our previous findings [15], which led us to hypothesize that neutralization of AT might prevent biofilm formation on mucosal tissue. Indeed, anti-AT specific human monoclonal IgG (MEDI4893 MedImmune, Fredricksburg, MD, USA pretreatment of ex vivo mucosa (1 h) completely abrogated biofilm formation by USA300 MRSA strain LAC and limited the strain’s epithelial cytotoxicity (Figure 4A), whereas c-IgG had no effect on biofilm or toxicity (Figure 4B). 

### 2.6. Rescue of Biofilm Defect with Exogenous AT

**Figure 5:**
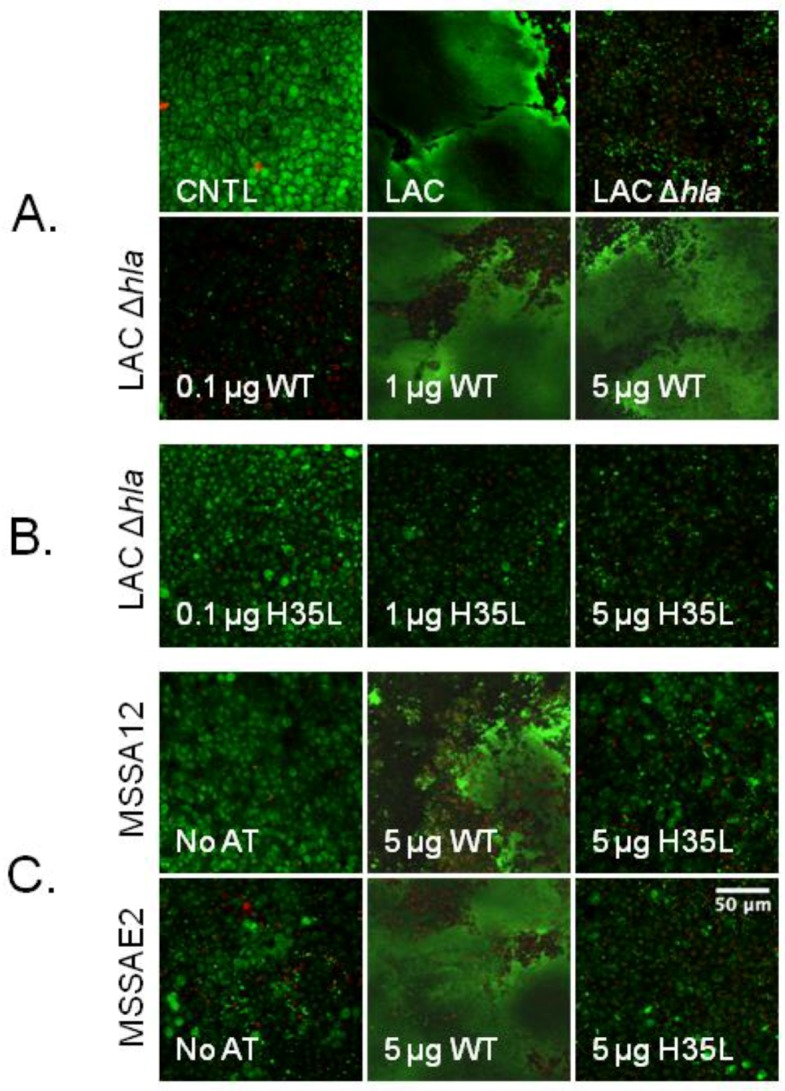
**Wild-type alpha-toxin rescues biofilm defect for MRSA LAC Δhla mutant and clinical isolates.** Porcine vaginal mucosa (PVM) explants were pretreated with 0.1–5 µg/explant of wild-type (WT) alpha-toxin (AT) or AT H35L for 10 min prior to infection. Three days post-infection, explants were stained with LIVE/DEAD and imaged by confocal laser scanning microscopy. (**A**) Uninfected control; explants infected with MRSA LAC show extensive biofilm formation, while explants infected with MRSA LAC Δhla exhibit very little to no biofilm. Explants pretreated with WT AT prior to addition of MRSA LAC Δhla exhibit biofilm rescue only at high doses. (**B**) Explants pretreated with AT H35L show no biofilm rescue at any dose. (**C**) WT AT rescues the biofilm defect of both MSSA12 and MSSAE2, while AT H35L does not. Scale bar equals 50 μm.

The biofilm defect of SA strain LAC Δ*hla* was rescued by pre-treatment of tissue explants with exogenous, purified WT AT (concentration 0.25 μg/μL), prior to bacterial inoculation in a dose-dependent manner. That is, AT enhanced biofilm production when applied at a dose of 1 or 5 μg/explant, but not at 0.1 μg/explant, which had no effect (Figure 5A). In contrast, no amount of the non-pore-forming AT H35L could rescue LAC Δ*hla*’s biofilm defect (Figure 5B). Similarly, 5 μg/explant of WT AT rescued the ex vivo biofilm defect of two clinical isolates, MSSA12 and MSSAE2, which possess the *hla* nonsense mutation (Figure 5C). 

**Figure 6:**
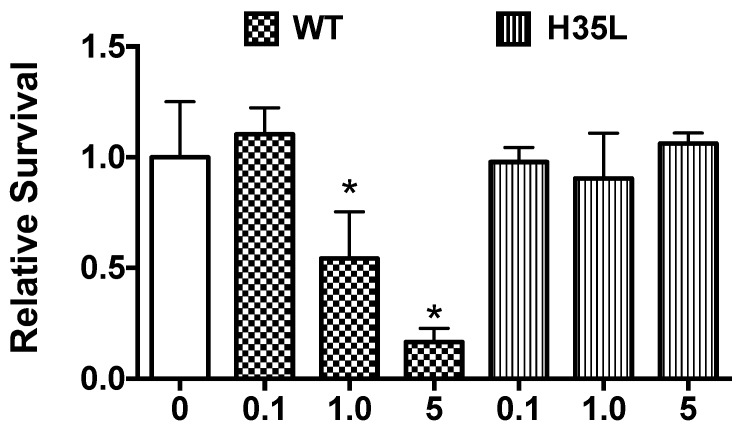
**Toxicity of wild-type and H35L mutant alpha-toxins.** Purified wild-type (WT) alpha-toxin (AT) or AT H35L mutant was added to porcine vaginal mucosa explants at varying doses and viability was determined by an MTT-based assay. WT AT was cytotoxic at higher concentrations (1 and 5 μg/explant), while the AT H35L mutant (non-pore forming) was non-toxic. Data (*n* = 3) are represented as mean ± SD; * denotes significance from non-AT treated control explants; *p* < 0.05 as determined by one-way analysis of variance followed by Dunnett’s multiple comparisons post-test.

We added WT AT and AT H35L separately at various doses to ex vivo PVM and assessed cytotoxicity to determine if the ability of exogenous AT to rescue the biofilm defect of AT-nonproducing SA strains corresponds with the cytotoxicity of AT for epithelial cells. As expected, the AT H35L was not toxic to PVM at any dose tested, whereas WT AT was toxic at 1 μg and 5 μg/explant, but not at 0.1 μg/explant (Figure 6). 

While we could recover up to approximately 10 ng/explant of AT from SA isolates growing on PVM for 72 h (Figure 1A,C), we require at least 1 μg purified WT AT/explant to rescue the biofilm defect (Figure 5). To understand why more exogenous AT is needed to support biofilm formation than what we can recover from tissue infected with AT-producing strains, we investigated the proportion of exogenous AT recoverable from PVM. To do so, we added WT AT to PVM explants and incubated for 24 h, and then analyzed recoverable AT by ELISA. The fraction of applied AT recovered from PVM explants after 24 h incubation was 0% when 0.1 μg AT was applied and only approximately 0.3% when either 1.0 μg or 5 μg were applied (Figure S2). These data indicate that the amount of AT recovered from PVM infected with SA is only a small fraction of the amount of AT being produced by the bacteria over time. 

## 3. Discussion

*S. aureus* is a common wound isolate, and AT exacerbates wound severity in dermonecrosis infection models [22,23]. However, the mechanisms by which AT contributes to wound pathology remains largely unknown. Biofilms play an important role in the infection process by creating a barrier that allows SA to evade the host immune system and resist antibiotics, thus interfering with wound healing and prolonging infection [4]. It is unknown whether AT’s role in promoting biofilm formation contributes to involvement in promoting wound infections and blocking wound healing. 

Therefore, we analyzed a collection of SA wound isolates to understand AT’s role in SA biofilm formation. These analyses included genetic background (PFGE type, MLST, and *spa* type) and *hla* sequencing. The dominance of ST8 and ST5 and the corresponding *spa* types (t008 and t002) among the present MRSA isolates was consistent with current trends in the United States [8,24,25,26] suggesting that our findings can be generalized. DNA sequencing of the AT-encoding gene *hla* in the MSSA isolates revealed that four out of seven (57%) of the isolates with an ex vivo biofilm score of <1 harbored a nonsense mutation at position 113 (Q113Stop). One of the remaining three isolates, MSSA 4, did not have the Q113Stop mutation but had multiple other nonsense mutations throughout the *hla* coding sequence (data not shown). A limitation of this study is the chosen sequencing region of *hla* (position 1 to 873 bp) spanned codon 113 but did not encompass sequence upstream of the codon region or the entire open reading frame. Therefore, we cannot make further conclusions related to genotype and AT production outside of the sequenced region. 

Among the MRSA and MSSA clinical wound isolates, ex vivo biofilm formation was strongly correlated with AT production, which is consistent with our previous findings in a clinical pneumonia MSSA isolate and its isogenic AT knockout derivative [15]. Interestingly, ex vivo and in vitro biofilm formation did not correlate; some strains were able to form biofilms in vitro but not ex vivo (e.g., MSSA D1, E1 and E2). Our data indicate that assessing biofilm formation on abiotic polystyrene surfaces may not be suitable for predicting SA biofilm formation on biological surfaces. Therefore, we propose that an analysis of ex vivo AT production will better predict in vivo mucosal biofilm formation. This proposal is supported by studies that determined that the presence of AT was highly predictive of the development of ventilator-associated pneumonia [27], a biofilm-associated infection [28]. 

To explore the role of AT in ex vivo biofilm formation further, we assessed the ability of the anti-AT mAb-MEDI4893* to inhibit SA biofilm formation. MEDI4893* completely abrogated MRSA LAC biofilm formation on ex vivo tissue. MEDI4893* prevents binding of AT to its cellular receptor ADAM10, effectively blocking pore formation [29]. While it is possible that MEDI4893* prevents biofilm by inhibiting another activity of AT, our data suggest that AT pore formation may be critical for AT to promote biofilm formation on epithelial tissues. Anti-AT antibodies have shown efficacy in the treatment and prevention of murine SA dermonecrosis and pneumonia [30,31]. Dosing animals with a panel of mAb anti-AT antibodies prior to or following infection increased survival, reduced bacterial burden in the lungs and kidneys, reduced lesion sizes, and enhanced the host immune response. The data presented here suggest that anti-AT antibodies may decrease SA infection severity by inhibiting SA biofilm formation. Additional work in vivo is required to determine if SA biofilms in a wound are reduced or absent when animals are dosed with anti-AT antibodies prior to or following infection.

Finally, we confirmed that AT is required for SA biofilm formation by adding exogenous AT to ex vivo explants prior to infection with SA strains (MSSAE2 and MSSA12) harboring the Q113Stop mutation in *hla* or MRSA LAC Δhla mutant. WT AT rescued the biofilm-forming defect of these strains, while the binding but non-pore-forming AT H35L mutant did not rescue at any dose tested. Additionally, WT AT only rescued at doses that were cytotoxic to the explants. 

Although our study strongly suggests a role for alpha toxin in mucosal biofilm formation there are limitations such as the potential contribution of other cytolytic toxins. The MRSA USA300 LAC strain has been characterized previously to possess genes that encode for other cytolytic toxins, such as gamma-toxins (HlgAB and HlgCB), leukocidins and Panton–Valentine leucocidin (PVL), which bind specific receptors on the cell surface in a species-specific manner [32,33]. Both PVL and HlgCB bind C5aR receptor with binding activity restricted to human and rabbit cells [33]. Additionally, once PVL binds C5aR the toxin is internalized, which is where part of the pore-forming process occurs [34]. Although our data clearly demonstrate a loss of ex vivo (porcine) mucosal biofilm formation in the MRSA LAC Δhla mutant, the production of these other cytolytic toxins by MRSA USA300 LAC or MRSA LAC Δhla mutant strains was not determined. Furthermore, whether the ex vivo porcine mucosal tissue produces C5aR which can bind other cytolytic toxins, i.e., PVL, and is sensitive to detect a contribution to biofilm formation is unknown at this time. 

The concentration-dependent biofilm-forming effect of AT was validated by our finding that approximately 0.3% of the total purified AT (1 μg or 5 μg) was recovered from explants after 24 h incubation (Figure S2). These data indicate that the amount of AT recovered from explants infected with the SA clinical wound isolates is only a small fraction of the total amount being produced (e.g., 5–10 ng/explant of AT recovered is closer to 1.67–3.3 μg/explant AT produced). This estimated amount of AT produced by the clinical SA wound isolates per explant is similar to the amount of purified AT required per explant to promote biofilm formation in the MRSA LAC Δhla mutant. These data confirm that AT is required for biofilm formation on ex vivo tissue and suggest that this phenotype is associated with AT’s ability to lyse underlying host cells. The lysis of host cells may supply SA with a critical source of nutrients, aid in SA adhesion, and induce the production of biofilm components. 

The potential benefits of neutralizing AT are 2-fold: (1) reduced biofilm formation, which should increase the opportunity for conventional antibiotics and host defense mechanisms to exert their effects, and (2) enhanced wound healing, since AT is cytotoxic to both epithelial and immune cells. Two published preclinical animal studies support the potential clinical benefits of neutralizing AT in a SA infected wound. One study determined that prophylactic treatment with anti-AT mAb-MEDI4893* in a SA skin wound murine infection model decreased wound size, bacterial burden, enhanced re-epithelialization and wound resolution compared to a control mAb [35]. Additionally, combined treatment with MEDI4893* and an antibiotic, compared to antibiotics alone, improved disease outcome and accelerated wound healing in an SA murine dermonecrosis model [36]. Together with these findings, our results suggest that the anti-AT mAb-MEDI4893* may effectively reduce SA biofilm formation on tissue and reduce epithelial toxicity and thus represents an alternative or enhanced strategy for preventing and treating biofilm-related SA wound infections.

## 4. Materials and Methods 

Bacteria: The 38 SA wound isolates included in the study were unique (by patient), deidentified clinical wound isolates that were collected without other selection criteria from the MVAMC clinical microbiology laboratory from May through July 2013. Some experiments used the USA 300 strain LAC, an SA abscess isolate from 2002 [37], or its isogenic Δ*hla* mutant (kind gift of Alex Horswill, University of Iowa) [12]. Prior to experimentation, tryptic soy agar containing 5% sheep blood (TSA-B, Beckton-Dickenson, Franklin Lakes, NJ, USA) was streaked with isolates from frozen glycerol stocks. On the afternoon prior to experimentation, Todd Hewitt broth (THB) (Becton-Dickenson) was inoculated from the TSA-B plate.

Pulsed-field gel electrophoresis, multi-locus sequence typing, and Spa typing. *Sma*I pulsed-field gel electrophoresis (PFGE) and determination of PFGE-based clonal groups was performed as described previously [38]. Isolates with a profile <80% similar to any reference strain were described as non-typeable. Genomic DNA was extracted and multi-locus sequence typing (MLST) performed as described previously [21]. Allelic designations and sequence types were assigned via the SA MLST database (http://saureus.mlst.net). Isolates were further typed by *spa* sequencing, with sequences classified according to the Ridom spa-typing database (http://spa.ridom.de/index.shtml).

*hla* PCR and sequencing. An 873-bp region (position 1 to 873) of the *hla* gene (960-bp) was amplified with the following primers: forward 5′-TGAAAACACGTATAGTCAGC-3′ (positions 1 to 21) and reverse 5′-CCAATTTGTTGAAGTCCAA-3′ (positions 873 to 857). The PCR product was sequenced and compared to sequences for strains MNPE (*hla* wt) and MN8 (*hla* Q113stop codon mutation) with ApE freeware. 

In vitro biofilm formation assay. In vitro biofilm was quantified by a crystal violet-staining method [20,39]. SA isolates were grown overnight in THB and then diluted 1:10 in 96-well microtiter plates (Corning 3595; Sigma, St. Louis, MO, USA) containing tryptic soy broth (TSB) supplemented with 1% glucose (Fluka, Ronkonkoma, NY, USA). Plates were incubated with gentle rocking at 37 °C for 24 h. Adherent cells and biofilm extracellular matrix were stained with aqueous 1% crystal violet (Sigma-Aldrich, St. Louis, MO, USA) for 1 h at room temperature. After staining, wells were rinsed gently until water ran clear. Stain was solubilized in 70% ethanol at room temperature for 2 h. Optical density was measured at 600 nm. Non-inoculated wells served as controls. Bacteria were divided into the four categories based on extent of biofilm formation modified according to Christensen et al.: non-biofilm forming (non-adherent, OD = 0), weakly biofilm forming (weakly adherent, OD > 0 to <0.24), strong biofilm forming (strongly adherent, OD ≥ 0.24 to 1) and very strong biofilm forming (very strongly adherent, OD > 1) [39]. 

Ex vivo porcine vaginal mucosa culture. We previously described an ex vivo biofilm infection model that uses porcine vaginal mucosa (PVM) explants as the substrate [19]. Briefly, explants of PVM from fresh tissue obtained from the Andrew Boss Laboratory of Meat Science, University of Minnesota, were obtained with a 5 mm biopsy punch. Explants were triple washed in serum and antibiotic-free RPMI 1640 (Gibco Life Technologies, Carlsbad, CA, USA) and then placed mucosal-side up on a 0.4-μm cell culture insert (BD Bioscience, San Jose, CA, USA) in 6-well plates containing fresh RPMI 1640 medium. For inoculation, stationary phase cultures were washed in RMPI 1640 medium and resuspended to approximately 5 × 10^8^ CFU/mL. PVM explants were inoculated with 2 µL of this suspension (1 × 10^6^ CFU/explant) and incubated with the mucosal surface continually exposed to air at 37 °C + 7% CO_2_ for 72 h (corresponds with mature biofilm formation by SA on PVM explants) [19]. For biofilm rescue experiments, AT or the variant cell binding but non-cytolytic AT H35L mutant (MedImmune, Fredricksburg, MD, USA) [31] were added to explants at the indicated doses for 10 min prior to inoculation of bacteria.

Ex vivo biofilm growth, imaging, and scoring. Following co-culture for the indicated times, PVM explants were stained with Filmtracer™ LIVE/DEAD^®^ stain (Molecular Probes, Invitrogen, Carlsbad, CA, USA) according to the manufacturer’s instructions and then examined by confocal laser scanning microscopy (CLSM, Zeiss Axioscope II). Images were captured at 63× original magnification with Lasersharp software and processed with ImageJ software. Explants were examined and scored independently by two blinded observers. We devised a semi-quantitative scoring system to capture the degree of biofilm formation (Figure 1): 0, no biofilm observed; 1+, >0 to 25% surface area coverage; 2+, 26% to 50% coverage; 3+, 51% to 75% coverage; and 4+, 76% to 100% coverage.

AT toxicity assay: Purified WT AT or the AT H35L mutant protein was added to PVM explants at varying doses and incubated at 37 °C + 7% CO_2_ for 24 h. Explants were triple washed, and viability was determined with the Cell Viability Determination Kit (Sigma-Aldrich), an MTT-based assay.

AT enzyme-linked immunosorbent assay. AT was quantified by sandwich enzyme-linked immunosorbent assay (ELISA), as previously described, using an anti-AT-specific human monoclonal IgG (MEDI4893*) (MedImmune, Fredricksburg, MD, USA) for capture and polyclonal rabbit anti-AT and peroxidase conjugated goat anti-rabbit IgG (Abcam, Cambridge, MA, USA) for detection [30]. The assay’s lower limit of detection was 60 pg/mL. 

AT recovery from PVM. WT AT was applied to PVM explants at varying doses, and explants were incubated for 24 h at 37 °C + 7% CO_2_. Explants were then vortex-mixed in PBS and the vortexed samples were analyzed for AT by ELISA (described previously).

Bacterial enumeration. Bacteria from PVM explants were enumerated by serial dilution plating of vortexed samples. Briefly, at 72 h post-inoculation, the PVM explants were transferred to tubes containing sterile PBS and vortex mixed to release bacteria. (Vortex mixing was equivalent to sonication for disrupting biofilm-associated bacteria (Figure S3.) Vortexed samples were plated onto TSA-B agar plates neat and serially diluted in PBS. Colonies were counted following overnight incubation at 37 °C + 7% CO_2_.

Neutralization of AT with anti-AT mAb MEDI4893* (MedImmune, Fredricksburg, MD, USA). MEDI4893* prevents binding of AT to its cellular receptor ADAM10, effectively blocking pore formation [29]. PVM explants, prepared as described above, were pretreated with 10 µg MEDI4893* or isotype control IgG (c-IgG) for 1 h at 37 °C. Explants were then inoculated with USA300 MRSA strain LAC and incubated, stained, and imaged as described above.

Statistical analysis: Data were analyzed by Student’s *t*-test or one-way ANOVA with Bonferroni’s post-test correction by means of GraphPad Prism 5 Software (GraphPad Software Inc., La Jolla, CA, USA). Data are representative of two or three experiments, with *n* = 3–8 per group per experiment. Spearman’s rank order was used to assess bi-isolate correlations, separately for 20 MSSA isolates and 18 MRSA isolates. Each isolate was examined in triplicate by two independent observers, for a total of six observations per isolate. The categorical interpretation of Spearman’s correlation coefficient (r_s_) for strength of correlation was as follows: 0.00–0.19, very weak; 0.20–0.39, weak; 0.40–0.59, moderate; 0.60–0.79, strong; and 0.80–1.0, very strong.

## Figures and Tables

**Table 1:** Summary of classifications of *Staphylococcus aureus* wound isolates.

Methicillin Phenotype	Isolate	PFGE USA Type ^a^	MLST ^b^	Spa ^c^	Q113Stop ^d^
MRSA	4	100	ST231	t002	ND
	5	100	ST5	t088	ND
	12	100	ST5	t002	ND
	13	100	ST5	t002	ND
	A2	100	ST5	t002	ND
	A3	100	ST5	t002	ND
	B1	100	ST5	t002	ND
	C1	100	ST5	t105	ND
	C2	100	ST5	t002	ND
	C3	100	ST5	t002	ND
	9	300	ST8	t008	ND
	10	300	ST8	t008	ND
	15	300	ST8	t008	ND
	16	300	ST8	t008	ND
	A1	300	ST8	t008	ND
	B2	300	ST8	t008	ND
	2	Non-typeable	ST5	t242	ND
	B3	Non-typeable	ST88	t11140	ND
MSSA	D3	100	ST231	t548	no
	D1	200	ST30	t012	yes
	1	200	ST30	t012	yes
	5	200	ST30	t1577	yes
	E2	200	ST30	t012	yes
	13	200	ST45	t015	no
	11	300	ST8	unknown	no
	8	400	ST1	t127	no
	9	400	ST1	t127	no
	2	600	ST45	t073	no
	3	900	ST15	t084	no
	E1	900	ST15	t084	no
	12	Non-typeable	ST30	t1577	yes
	E3	Non-typeable	ST30	t012	yes
	4	Non-typeable	ST39	t2271	no
	10	Non-typeable	ST5	t010	no
	F2	Non-typeable	ST5	t002	no
	6	Non-typeable	ST8	unknown	no
	F3	Non-typeable	ST97	t521	no
	D2	non-typeable	ST97	t267	no

^a^ PFGE, pulsed-field gel electrophoresis. USA type, as assigned by comparison with CDC reference strains. Ten of 18 MRSA isolates (56%) were USA100, 6/18 (33%) were USA300 and 2/18 (11%) were non-typeable. The majority of the MSSA isolates (8/20, 40%) were non-typeable, 5/20 (25%) were USA200 and the remainder were diverse; ^b^ MLST, multi-locus sequence type. Of the MRSA isolates, 10/18 (56%) were ST5 and 6/18 (33%) were ST8. Of the MSSA isolates, 6/20 (30%) were ST30, including 4 USA200 isolates and two that were non-typeable by PFGE; the other 14 (70%) were diverse; ^c^ Spa sequencing; ^d^ Six of 20 MSSA isolates had a stop codon mutation at position 113 in hla, which is; commonly associated with PFGE USA200/ST30; ND, not determined.

## Supplementary Materials

The following are available online at http://www.mdpi.com/2072-6651/10/4/157/s1, Figure S1: Total CFU recovered from explants infected with different *S. aureus* isolates were similar, Figure S2: Less than 1% of exogenous alpha-toxin is recovered, Figure S3: Recovery of biofilm CFU is similar when explants are vortex mixed or sonicated followed by vortex mixing.
