# Supplementary material for: Alpha-Toxin Contributes to Biofilm Formation among Staphylococcus aureus Wound Isolates
Source: Toxins (Basel). 2018 Apr 16;10(4):157. doi: 10.3390/toxins10040157 (PMC5923323; doi:10.3390/toxins10040157)
Supplement: Supplementary file 1 [file toxins-10-00157-s001.pdf]

Supplementary Materials: Alpha-Toxin Contributes to Biofilm Formation among *Staphylococcus aureus* Wound Isolates

Michele J. Anderson, Emily Schaaf, Laura M. Breshears, Heidi W. Wallis, James R. Johnson, Christine Tkaczyk, Bret R. Sellman, Jisun Sun and Marnie L. Peterson

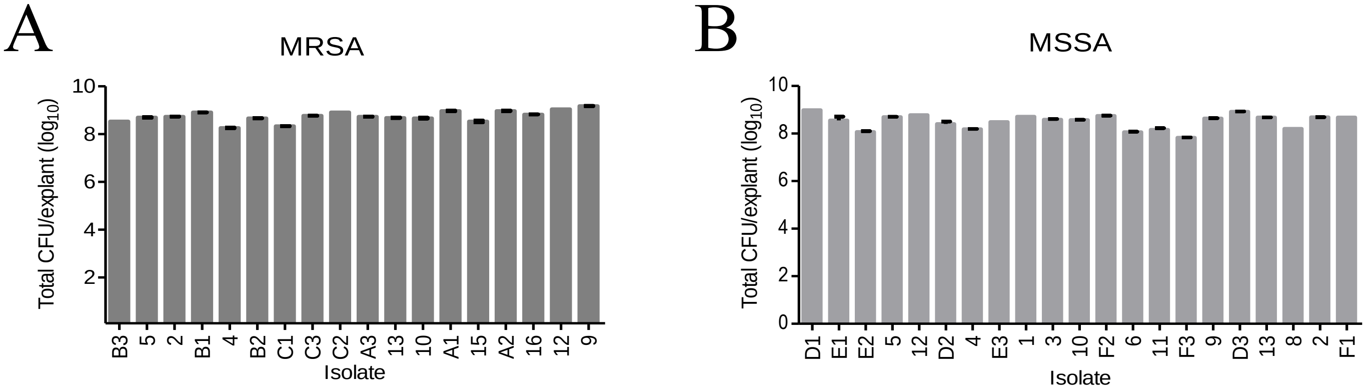

Figure. S1. Total CFU recovered from explants infected with different *S. aureus* isolates were similar. Explants were infected and cultured for 72 h then transferred to 1.7 mL snap cap tubes containing 250 µl of PBS for CFU recovery. Tubes were vortex mixed for 4 min on medium-high setting, serially diluted in PBS and plated on TSA-B to enumerate CFU. All isolates grew up to a similar density, near 1 x 10^8 log_{10} CFU/explant.

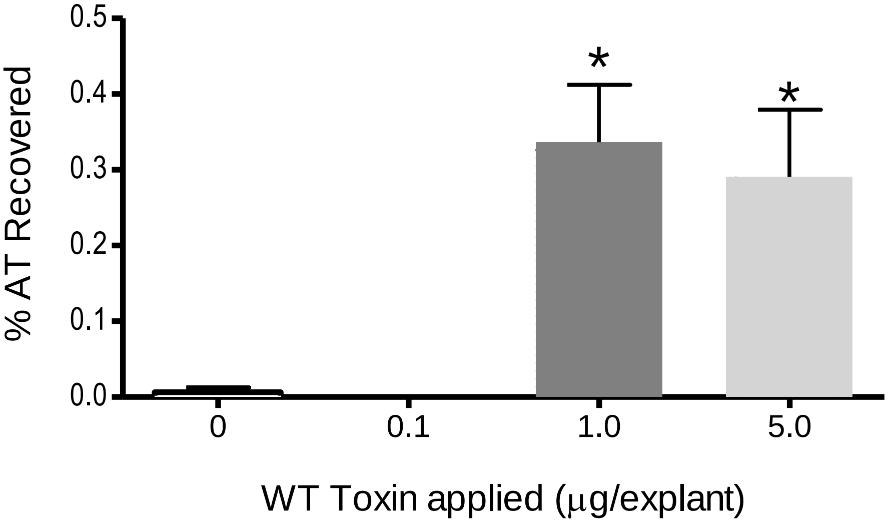

Figure S2. Less than 1% of exogenous alpha-toxin is recovered. Porcine vaginal mucosa (PVM) explants were treated with 0.1 – 5 µg/explant with wild-type (WT) alpha-toxin (AT) and incubated for 24 h. Explants were vortex mixed and the vortexates analyzed for AT recovery via ELISA. Only ~0.3% of AT was recovered from the higher applied doses. Data (n=3) are represented as mean ± SD; * denotes significance from non-AT treated control explants; $p<0.05$ as determined by one-way analysis of variance followed by Dunnett's multiple comparisons post-test.

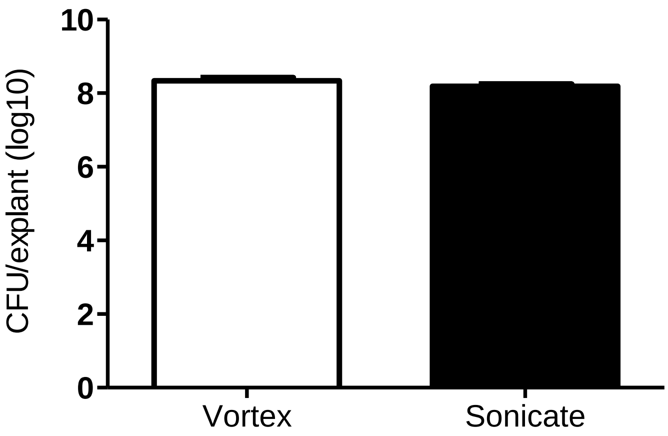

Figure. S3. Recovery of biofilm CFU is similar when explants are vortex mixed or sonicated followed by vortex mixing. Explants were infected with *S. aureus* and cultured for 72 h then transferred to 1.7 mL snap cap tubes containing 250 µl of PBS for CFU recovery. Tubes were either vortex mixed for 4 min on medium-high setting or sonicated in a water bath for 5 min followed by vortex mixing for 4 min on medium-high setting. No statistical difference was observed, n=4, p=0.19.
